# Insulin‐like growth factor 2 mRNA‐binding protein 2‐regulated alternative splicing of nuclear factor 1 C‐type causes excessive granulosa cell proliferation in polycystic ovary syndrome

**DOI:** 10.1111/cpr.13216

**Published:** 2022-03-16

**Authors:** Feiyan Zhao, Liang Wu, Qin Wang, Xuehan Zhao, Tong Chen, Chenghong Yin, Long Yan, Xiaokui Yang

**Affiliations:** ^1^ Department of Human Reproductive Medicine Beijing Obstetrics and Gynecology Hospital, Capital Medical University Beijing People's Republic of China; ^2^ Department of Human Reproductive Medicine Beijing Maternal and Child Health Care Hospital Beijing People's Republic of China; ^3^ State Key Laboratory of Stem Cell and Reproductive Biology Institute of Zoology, Chinese Academy of Sciences Beijing People's Republic of China; ^4^ Reproductive Medical Center The First Affiliated Hospital of Zhengzhou University Zhengzhou Henan People's Republic of China; ^5^ Henan Key Laboratory of Reproduction and Genetics The First Affiliated Hospital of Zhengzhou University Zhengzhou Henan People's Republic of China; ^6^ Department of Internal Medicine Beijing Obstetrics and Gynecology Hospital, Capital Medical University Beijing People's Republic of China; ^7^ University of Chinese Academy of Sciences Beijing People's Republic of China; ^8^ Department for Stem Cell and Regeneration Chinese Academy of Sciences Beijing People's Republic of China; ^9^ Beijing Institute for Stem Cell and Regenerative Medicine Beijing People's Republic of China

## Abstract

**Objectives:**

Polycystic ovary syndrome (PCOS) is a common reproductive endocrine disorder. Insulin‐like growth factor 2 mRNA‐binding protein 2 (IGF2BP2) serves as an HMGA2 target gene to promote the proliferation of granulosa cells (GCs). However, it is still unclear whether IGF2BP2 participates in the pathogenesis of PCOS as RNA binding protein (RBP). In this study, we aimed to elucidate IGF2BP2‐interacting transcripts, global transcriptome together with alternative splicing in GCs to eventually uncover potential mechanisms of PCOS pathogenesis.

**Materials and Methods:**

The expression of IGF2BP2 in GCs from PCOS patients was detected using quantitative reverse transcription PCR (RT‐qPCR) and western blot. We captured IGF2BP2‐interacting transcripts, global transcriptome together with alternative splicing by RNA immunoprecipitation sequencing (RIP‐seq) and RNA sequencing (RNA‐seq). KGN cells transfected with IGF2BP2 overexpressing plasmids and nuclear factor 1 C‐type (NFIC) siRNAs, were applied to CCK‐8, EdU and TUNEL assays.

**Results:**

IGF2BP2 was highly expressed in GCs from PCOS patients. As an RBP, it preferentially bound to the 3′and 5′UTRs of mRNAs with GGAC motif and a newly found GAAG motif. The overexpression of IGF2BP2 changed the transcriptome profile of KGN cells. IGF2BP2 functioned to regulate alternative splicing events and promote cell proliferation through inhibiting exon skipping events of NFIC.

**Conclusion:**

In conclusion, we demonstrated that IGF2BP2 promotes GC proliferation via regulating alternative splicing of NFIC in PCOS. The findings help to better understand the roles of IGF2BP2 in the pathogenesis of PCOS.

## Graphical abstract

Polycystic ovary syndrome (PCOS) is a common reproductive endocrine disorder, affecting approximately 10% of women of reproductive age. Insulin‐like growth factor 2 mRNA‐binding protein 2 (IGF2BP2) reportedly functions as an RNA‐binding proteins (RBPs) that regulates a series of target genes by binding RNA transcripts. However, it is still unclear whether IGF2BP2 participates in pathogenesis of PCOS. In this study, we detected expression of IGF2BP2 in granulosa cells (GCs) from PCOS patients and captured IGF2BP2‐interacting transcripts, global transcriptome together with alternative splicing by RIP‐seq and RNA‐seq in immortalized human GCs (KGN cells). Our results show that IGF2BP2 is overexpressed in GCs from PCOS patients. As an RBP, it preferentially binds to the 3′and 5′UTRs of mRNAs with GGAC motif and a newly found GAAG motif. Moreover, overexpression of IGF2BP2 can promote cell proliferation through regulating alternative splicing of NFIC in KGN cells. In conclusion, we provide a transcriptome‐wide analysis that demonstrates the role played by IGF2BP2 in the pathogenesis of PCOS. The findings of this study will help us to better understand the aetiology of PCOS from the perspective of IGF2BP2 mediated alternative splicing as well as suggest opportunities for future personal therapeutic guidance for PCOS.

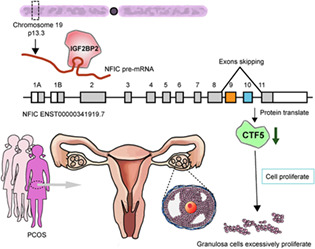

## 1 INTRODUCTION

The ovary, serving as an important reproductive organ in females, performs a variety of functions including generating oocytes, secreting sex hormones and maintaining estrous cycles.^1^ Polycystic ovary syndrome (PCOS) is a heterogeneous disorder whose hormonal disbalance induces clinical manifestations such as numerous cysts, irregular menstrual cycles and even infertility.^2^ As a syndrome, the pathological mechanisms underlying PCOS are complicated since the clinical features are anfractuous and the etiologies differ among individuals.^3^ To date, factors including genetics, chronic inflammation, oxidative stress, lifestyle as well as environmental pollution have been identified to be associated with PCOS occurrence and progression. The ovarian granulosa cells (GCs), which serve as ovarian somatic cells that surround oocytes, can interact with the developing oocyte and are therefore essential for folliculogenesis.^4^ Previous reports have confirmed that aberrant GC proliferation and differentiation disrupt normal ‘dialogue’ between oocyte and GCs particularly in early growing follicles of PCOS women.^5^

Recently, RNA‐binding proteins (RBPs) are gaining recognition for their importance in unveiling the mechanistic processes of dysfunctional GCs behind the pathogenesis of PCOS.^6^, ^7^, ^8^ Alternative splicing (AS), allowing the generation of more than one unique mRNA species from a single gene, is an important mechanism of gene expression, which regulates tissue identity and critical biological processes. The different AS isoforms might affect mRNA stability, localization, or translation.^9^ Furthermore, some splicing mRNA isoforms could change the reading frame, resulting in producing different protein isoforms with diverse functions and/or localizations. Increasing evidence indicates that RBPs can promote exon inclusion or skipping by binding to splicing regulatory elements.^10^, ^11^ Changes in the levels and activity of RBPs may lead to AS dysregulation and the production of diverse aberrant transcripts that might contribute to some human diseases. A previous study demonstrated two alternative splice variants of the androgen receptor were associated with remarkable hyperandrogenism and abnormal folliculogenesis in 62% of patients with PCOS.^12^

Insulin‐like growth factor 2 mRNA‐binding protein 2 (IGF2BP2), also known as IMP2 or VICKZ2, is a member of the IGF2 mRNA‐binding protein family and encoded by the IGF2BP2 gene, which is located on chromosome 3q27.^13^, ^14^ IGF2BP2 is associated with type 2 diabetes mellitus (T2DM) and obesity and has been reported to be involved in insulin resistance, lipid metabolism and tumorigenesis.^15^, ^16^ Previous studies have reported that IGF2BPs (IMPs) such as IGF2BP2 are expressed in male and female gonadal cells at embryonic day 12.5 (E12.5) in mice, and IGF2BP1, IGF2BP2 and IGF2BP3 are present in resting and growing oocytes as well as in the GCs of mature mouse and human ovaries.^17^ It has been recently reported that IGF2BP2 is highly expressed in the GCs of women with PCOS and participates in the high mobility group AT hook 2 (HMGA2)/IMP2 pathway to promote the proliferation of GCs.^8^ However, the comprehensive role played by IGF2BP2 in the pathogenesis of PCOS remains poorly understood.

In this study, we obtained IGF2BP2‐regulated transcriptomes in the IGF2BP2‐overexpression (OE) immortalized human GCs (KGN cells) by RNA sequencing (RNA‐seq). We also performed RNA‐immunoprecipitation (IP) sequencing (RIP‐seq) to comprehensively identify the mRNAs which are associated with IGF2BP2. Integrated analysis revealed that IGF2BP2 binding could regulate a variety of AS events in KGN cells and IGF2BP2‐mediated nuclear factor 1 C‐type (NFIC) AS promoted cell proliferation in PCOS.

## 2 MATERIALS AND METHODS

### 2.1 Study population

A total of 40 women were enrolled in the study in the Department of Human Reproductive Medicine, Beijing Obstetrics and Gynaecology Hospital from 1 January 2019 to 31 January 2021. They routinely signed informed consent after being fully informed of subsequent data collection for further study. Women with PCOS were selected in accordance with the Rotterdam criteria.^18^ The study was approved by the Ethics Committee of the Beijing Obstetrics and Gynaecology Hospital, Capital Medical University.

### 2.2 Preparation of human GCs

Human GCs were isolated from follicular fluid aspirates obtained at oocyte retrieval as described by Shi et al.^19^ Briefly, follicular fluid from each patient was centrifuged at 400 *× g* for 10 min, and the layers of GCs with the red blood cell pellet were resuspended. After shaking at 200 rpm for 20 min at 37°C, the cell suspensions were layered on 8.0 ml Ficoll‐Paque Plus (GE Healthcare) and centrifuged at 600 *× g* for 20 min. GCs at the interface were harvested and washed three times with 10 ml Dulbecco's modified Eagle medium (DMEM)/nutrient mixture F‐12 Ham (DMEM/F‐12) supplemented with 10% foetal bovine serum, 100 U/ml penicillin, 100 μg/ml streptomycin sulfate, and 1× GlutaMAX (Invitrogen). After final centrifugation for 5 min at 600 *× g*, the cells were prepared for the following RNA and protein extraction.

### 2.3 TUNEL assay

The apoptosis assessment was performed by TUNEL BrightRed Apoptosis Detection Kit (Vazyme) following the manufacturer's instructions. Briefly, cells climbing to the carry sheet glass were washed in PBS, fixed with 4% PFA and treated by 0.02% TritonX‐100. The cell slides were then incubated for 60 min at 37°C in TUNEL reaction solution (5:1 Label Solution: Enzyme Solution). The nuclei were finally stained with DAPI for 20 min at RT. The slides were washed by PBS three times and mounted with anti‐fade mounting medium. The image analysis was performed using ZEN software on at least three sections that were randomly selected from three separate experiments.

### 2.4 Statistical analysis

All data were reported as mean ± SEM. For normally distributed data, the parametric testing was used. For parametric testing between two groups, an unpaired Student *t*‐test was used. For non‐normally distributed data, Mann–Whitney *U*‐test was used. For the comparison of multiple‐groups, non‐paired one‐way ANOVA was used for nonpaired nonparametric data. Significance was evaluated using data from at least three independent experiments, with *P < 0.05, **P < 0.01, ***P < 0.001.

## 3 RESULTS

### 3.1 IGF2BP2 is highly expressed in GCs from PCOS patients and can increases cell viability and proliferation

**FIGURE 1:**
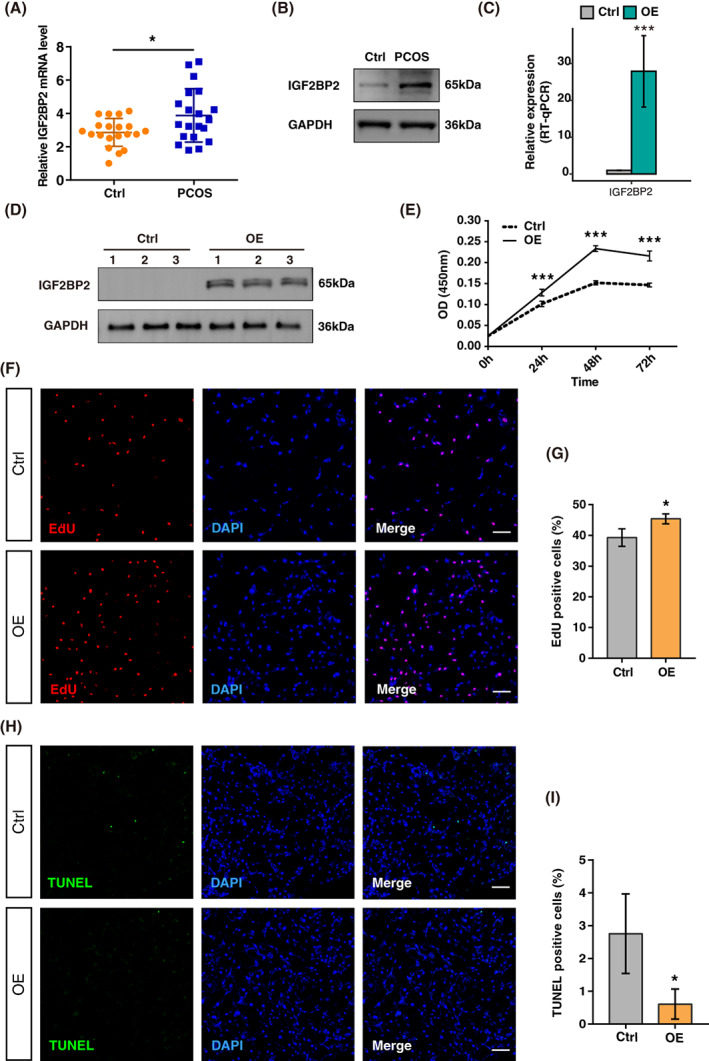
Upregulated insulin‐like growth factor 2 mRNA‐binding protein 2 (IGF2BP2) in granulosa cells (GCs) from polycystic ovary syndrome (PCOS) patients increases cell viability and proliferation. (A) IGF2BP2 mRNA detection by RT‐qPCR in GCs from Control and PCOS groups. Data were normalized to glyceraldehyde 3‐phosphate dehydrogenase (GAPDH). Control: *n* = 20; PCOS: *n* = 20. *P < 0.05. (B) IGF2BP2 protein detection by western blot in GCs from PCOS and Control women. (C) RT‐qPCR showing expression of IGF2BP2 mRNA in IGF2BP2 overexpression (OE) KGN cells. ***P < 0.001. (D) IGF2BP2 protein detection by western blot in IGF2BP2 OE KGN cells. Experiments were performed in triplicate. (E) KGN cells were transfected with vector control or IGF2BP2 OE plasmids for 24, 48, or 72 h, and cell viability was determined by the CCK‐8 assay. ***P < 0.001. (F) EdU staining of IGF2BP2 OE cells. Nuclei were stained by using DAPI. EdU positive cells, red; cell nuclei, blue; the data shown were representative of three independent experiments with similar results. Scale bar, 100 μm. (G) Statistics of EdU positive cells quantified by counting the cells with fluorescent signal using the software Image J. Experiments were performed in triplicate. *P < 0.05. (H) Apoptosis was reflected by TUNEL staining of the cells from the indicated groups. Representative images of the apoptotic cells were shown. Scale bar, 100 μm. (I) Statistics of TUNEL positive cells quantified by counting the cells with fluorescent signal using the software Image J. *P < 0.05. Ctrl, control. OE, IGF2BP2 overexpression. Error bars represent the mean ± SEM

To investigate the potential involvement of IGF2BP2 in the progress of PCOS, we analysed IGF2BP2 expression in a total of 40 GC samples from 20 PCOS patients and 20 controls who underwent in vitro fertilization‐embryo transfer/intracytoplasmic sperm injection (IVF/ICSI‐ET) (Figure S1). Clinical features were determined in Table 1. Basal testosterone was higher as well as antral follicle count was significantly much more in PCOS patients compared to normal women. Interestingly, IGF2BP2 was upregulated in both transcriptional and protein levels in GCs of PCOS patients (Figure 1A,B), suggesting it was an important regulator to modulate the pathogenesis of PCOS. Generally, increased GC proliferation is recognized as a key factor that underlies aberrant follicle maturation in PCOS.^20^ To determine if the high expression of IGF2BP2 promotes GC viability and/or proliferation, we conducted CCK‐8 and EdU assay, respectively. KGN cells were transfected with IGF2BP2 overexpressing plasmid, and the overexpression of IGF2BP2 was validated at mRNA and protein levels (Figure 1C,D). Cell viability and EdU positive ratio tended to be higher in KGN cells overexpressing IGF2BP2 (Figure 1E–G). Additionally, apoptosis cells testified by TUNEL assay were significantly decreased in this group (Figure 1H,I). Altogether, we demonstrated that IGF2BP2 was upregulated in GCs of PCOS patients and the overexpression of IGF2BP2 robustly increased cell viability and proliferation.

**TABLE 1:** General characteristics of the normal women control group and PCOS group

Characteristics	Control group (*n* = 20)	PCOS group (*n* = 20)	*P* value
Age, years	32.45 ± 4.25	30.35 ± 3.17	ns^a^
BMI, kg/m^2^	23.66 ± 2.86	25.88 ± 4.84	ns^a^
Basal FSH, IU/L	7.11 ± 1.10	5.76 ± 1.33	0.015^b^
Basal LH, IU/L	3.44 ± 1.22	5.08 ± 2.67	ns^b^
Basal E_2_, pg/ml	34.71 ± 12.07	33.63 ± 7.45	ns^b^
Basal T, nmol/ml	1.36 ± 0.31	2.37 ± 0.61	<0.0001^b^
Antral follicle count	11.75 ± 2.88	22.20 ± 3.46	<0.0001^a^

*Note*: Values are presented as mean ± standard deviation.

Abbreviations: BMI, body mass index; E_2_, estradiol; FSH, follicle‐stimulating hormone; LH, luteinizing hormone; PCOS, polycystic ovary syndrome; T, testosterone.

^a^ Statistical differences were calculated using Student *t*‐test.

^b^ Statistical differences were calculated using Mann–Whitney *U*‐test.

### 3.2 IGF2BP2 selectively regulates the expression of genes in inflammatory response and cell proliferation

**FIGURE 2:**
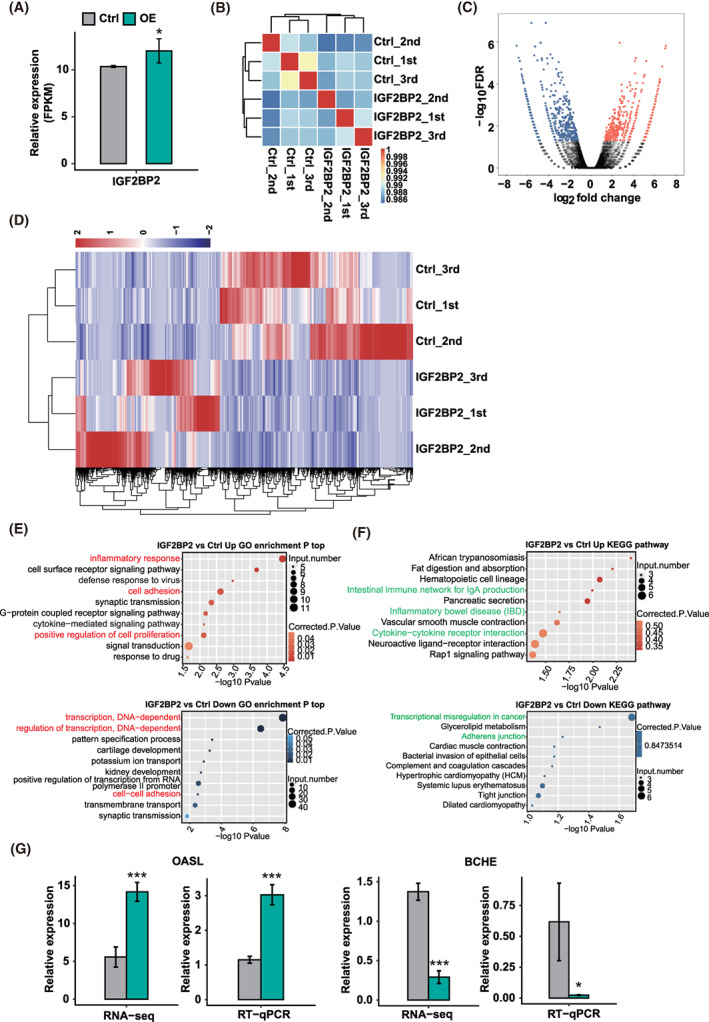
RNA‐seq analysis of insulin‐like growth factor 2 mRNA‐binding protein 2 (IGF2BP2) regulated transcriptome profile in KGN cells. (A) IGF2BP2 expression quantified by RNA‐seq data. Fragments per kilobase of transcript per million fragments mapped (FPKM) values were calculated following the described in Section 2. Error bars represent the mean ± SEM. *P < 0.05. (B) Heat map showing a hierarchically clustered Pearson's correlation matrix resulting from the comparison between transcript expression levels for control and IGF2BP2‐overexpressed samples. (C) Identification of IGF2BP2‐regulated genes. Volcano plot shows up‐ and down‐regulated genes labelled in red and blue, respectively. (D) Hierarchical clustering of differentially expressed genes (DEGs) in control and IGF2BP2‐overexpressed samples. FPKM values were log2‐transformed and then median‐centred by each gene. (E) The top 10 GO biological processes of IGF2BP2 up‐ and down‐regulated genes. (F) The top 10 KEGG pathways of IGF2BP2 up‐ and down‐regulated genes. (G) The relative expression of DEGs and qPCR validation. Error bars represent the mean ± SEM. ***P < 0.001, *P < 0.05

To explore the mechanisms of IGF2BP2, the transcriptome profiles of IGF2BP2 OE cells and control cells were detected by RNA‐seq. A total of six RNA‐seq libraries were constructed and sequenced for IGF2BP2 OE and control cells, with three biological replicates for each group (IGF2BP2_1st, IGF2BP2_2nd, IGF2BP2_3rd, Ctrl_1st, Ctrl_2nd, Ctrl_3rd). After removing sequence adaptors and low‐quality reads, the clean reads were mapped to the human genome GRCh38 using TopHat2: approximately 93.8% of the reads aligned and 87.37% were uniquely mapped in triplicates of IGF2BP2 OE (Table S2). Then, we calculated the gene expression using these uniquely mapped reads. Fragments per kilobase of transcript per million fragments mapped (FPKM) were calculated by an in‐house pipeline and used to represent the levels of gene expression. The results showed 17,257 genes expressed (FPKM >0) and 9890 genes expressed at level of FPKM >1 in at least one sample (Table S3). FPKM for IGF2BP2 further supported that this gene was effectively overexpressed in KGN cells (Figure 2A). A correlation matrix was calculated based on the FPKM values of expressed genes in all six samples. There was a high Pearson's correlation value between IGF2BP2 OE and the control (more than 0.986), which indicated the similar expression of most genes. However, unsupervised hierarchical clustering of the correlation matrix showed a clear separation of IGF2BP2 OE and control samples, with three biological replicates in a cluster (Figure 2B). To further compare the gene expression profiles, we identified the differentially expressed genes (DEGs) between the IGF2BP2 OE and control cells. There were 1141 DEGs in the RNA‐seq data in groups overexpressing IGF2BP2 (Figure 2C), of which 488 were upregulated and 653 were downregulated (following the criteria of Fold change [FC] ≥2 or ≤0.5 and FDR <0.05). Moreover, the hierarchical clustering of normalized FPKM values of DEGs showed a clear separation of the IGF2BP2 OE and control samples and a high consistency for the three replicate data sets (Figure 2D). These results illustrated that IGF2BP2 OE significantly changed the transcript expression level of a set of genes.

To reveal the potential roles of these DEGs, we subsequently examined GO enrichment analysis of the DEGs regulated by IGF2BP2 (Figure 2E). IGF2BP2‐upregulated genes were mainly associated with processes involving inflammatory response, cell surface receptor signalling pathway and positive regulation of cell proliferation. The IGF2BP2‐downregulated genes were mainly involved in transcription, DNA‐dependent regulation of transcription, cell–cell adhesion. The top 10 KEGG pathways associated with IGF2BP2‐upregulated genes included the intestinal immune network for IgA production, inflammatory bowel disease and cytokine‐cytokine receptor interaction; while those that were downregulated by IGF2BP2 were associated with transcriptional misregulation in cancer (Figure 2F). Both the upregulated and downregulated genes were enriched in terms related to inflammatory, cell proliferation and apoptosis.

To verify the sequencing data of expression of these DEGs, qPCR was conducted to quantify the changes in mRNA levels of these genes. Five DEGs associated with inflammatory were randomly selected for qPCR analysis, including OASL, BCHE, THEMIS2, FAM133B, STAB2 (Figure 2G; Figure S2). All selected DEGs were annotated in the GO or KEGG analysis, and the FPKM of these genes showed similar tendency in at least one sample. The results indicated that the selected DEGs showed a significant increase of OASL and THEMIS2 or decrease of BCHE, FAM133B and STAB after IGF2BP2 OE in KGN cells, which was in agreement with the RNA‐seq analysis.

### 3.3 IGF2BP2 preferentially binds to 3′ and 5′UTRs of mRNAs through GGAC and GAAG motifs

**FIGURE 3:**
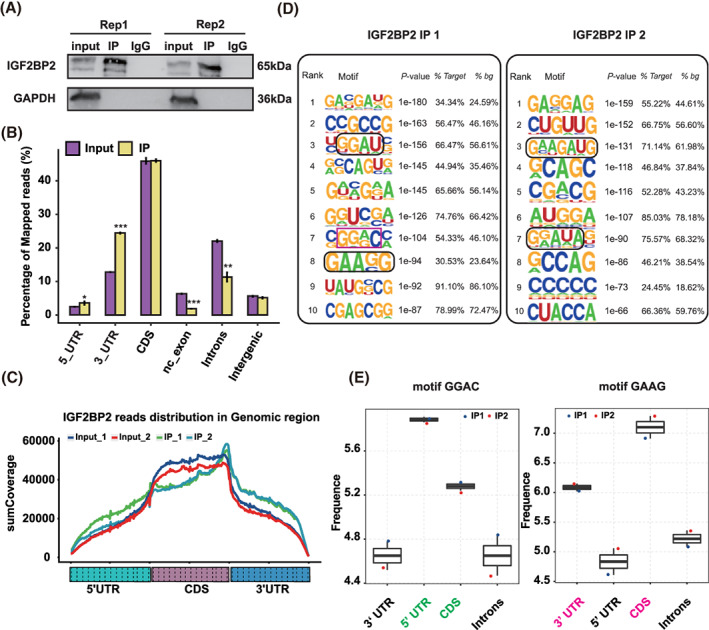
RNA immunoprecipitation sequencing analysis of insulin‐like growth factor 2 mRNA‐binding protein 2's (IGF2BP2) binding profile and binding motifs. (A) IGF2BP2 protein detection by western blot in KGN cells. (B) Read distribution across the reference genome. Error bars represent the mean ± SEM. *P < 0.05, **P < 0.01, ***P < 0.001. (C) The peak reads density for 5′UTR, CDS and 3′UTR for input and IGF2BP2 IP samples. (D) Motif analysis using the software HOMER showing the top 10 preferred binding motifs of IGF2BP2. (E) The binding sites of the GGAC and GAAG binding motifs

As previously reported, IGF2BP2 plays an important role in regulating AS.^21^ To better understand the roles of IGF2BP2 in the pathogenesis of PCOS, the IGF2BP2‐interacting transcripts were confirmed by RIP‐seq analysis in KGN cells. We were able to find abundant IGF2BP2 quantities in the immunoprecipitate fraction of KGN IGF2BP2‐overexpressing cells in two replicates (Figure 3A). We sequenced the cDNA libraries from the RNA inputs and the IGF2BP2 fraction. After removing the adaptor sequences and filtering out low‐quality reads, we obtained a total of 44,098,992 and 49,754,547 reads from each of the IGF2BP2 immunoprecipitates repetitions, and 58,215,977 and 60,099,967 reads from the two RNA input repetitions (Table S4). We mapped these four groups of reads to the human GRCH38 genome using Tophat2,^22^ about 57% aligned. From the mapping data, 6.3% and 4.9% of the total mapped reads were uniquely mapped in the immunoprecipitates. About 3.4% of the total mapped reads were uniquely mapped in the input groups (Table S4).

We plotted the distribution of uniquely mapped IGF2BP2 reads across reference genomic regions and found that the RIP‐seq reads were highly enriched in coding sequence (CDS) and 3′UTR regions (Figure 3B). And consistent with the previously reported ability of IGF2BP2 in regulating mRNA stability and translation, the intronic regions comprised a small percentage reads of the whole distribution.^23^ Furthermore, our analysis also revealed that the fractions of clean reads that mapped to the 3′ and 5′UTRs were significantly higher in the IGF2BP2 immunoprecipitates compared to the RNA input and more IGF2BP2‐binding peaks were observed in CDS than in intronic regions (Figure 3B,C). Overall, the immunoprecipitates showed that IGFBP2 mainly bound to 3′ and 5′UTRs of mRNAs.

We also searched for overrepresented motifs in the IGF2BP2‐binding peaks using the software Homer (http://homer.salk.edu/homer/motif/index.html). The GA‐rich motif was among the top 10 IGF2BP2‐binding motifs in two independent experiments (Figure 3D). It was reported that IGF2BPs, acting as a distinct family of *N* ^6^‐methyladenosine (m^6^A) readers, are able to recognize the GG (m^6^A) sequence to target mRNA transcripts. And in agreement with previous studies,^24^ we confirmed that GGAC was an enriched binding motif. Importantly, we found a novel motif GAAG, ranking eighth (IP1) and third (IP2) in the two immunoprecipitate experiments, respectively (Figure 3D). Moreover, in terms of the RNA binding, we noted that the GGAC motif was enriched at 5′ UTR and the GAAG motif was enriched in 3′UTR (Figure 3E). Taken together, our analysis on the distribution of reads indicated that IGF2BP2 preferentially bound to the 3′ and 5′UTRs due to the existence of GGAC and GAAG binding motifs.

### 3.4 Most of the DEGs are not regulated by direct binding to IGF2BP2

**FIGURE 4:**
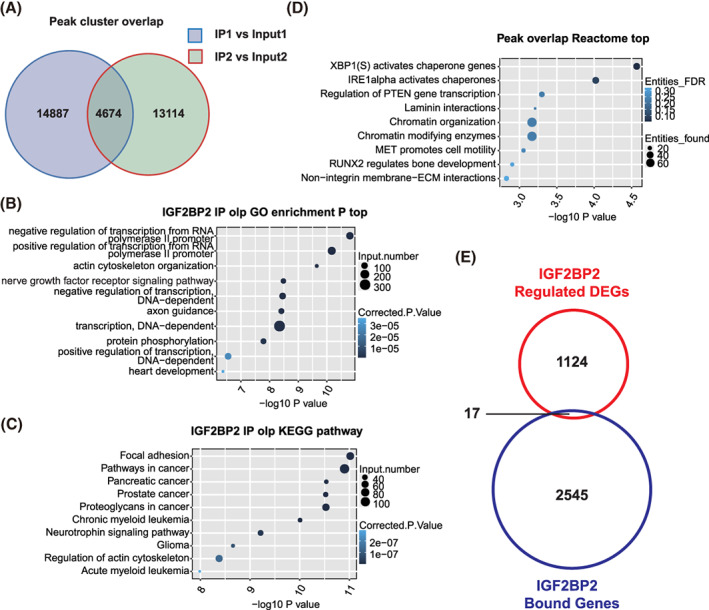
Differentially expressed genes (DEGs) regulated by insulin‐like growth factor 2 mRNA‐binding protein 2 (IGF2BP2) overexpression showed no direct bindings of IGF2BP2. (A) Venn diagram showing the overlap between peaks in two RNA immunoprecipitation sequencing (RIP‐seq) sample replicates. (B) Bubble plot showing the top 10 biological processes associated with IGF2BP2‐bound genes using a GO enrichment analysis. (C) Bubble plot showing the top 10 biological processes associated with IGF2BP2‐bound genes using a KEGG enrichment analysis. (D) Bubble plot showing the top 10 pathways associated with IGF2BP2‐bound genes using a reactome enrichment analysis. (E) Venn diagram showing genes overlapping between RIP‐seq and DEGs

In order to validate the reliable IGF2BP2‐bound genes, we called IGF2BP2‐bound peaks using three different methods, namely ABlife, Piranha and CIMS. The number of overlapped peaks was calculated using ABlife. A total of 4674 peak clusters consistently and repeatedly overlapped in the two RIP‐seq sample replicates (Figure 4A). We then performed GO and KEGG enrichment analysis to further examine the potential biological roles of these IGF2BP2‐bound genes (Figure 4B,C). The top 10 biological processes GO‐term enrichment demonstrated that IGF2BP2‐bound genes were associated with the regulation of transcription from RNA polymerase II promoter, the actin cytoskeleton organization and the nerve growth factor signalling pathway. Analogously, the enriched KEGG pathways included focal adhesion, pancreatic cancer and prostate cancer. Additionally, the reactome analysis showed that IGF2BP2‐bound RNAs were mainly associated with XBP1(s)‐activated chaperone genes and IRE1 alpha‐activated chaperones (Figure 4D). To explore whether IGF2BP2 altered its DEGs by directly binding them as RBP, we analysed the RNA‐seq and RIP‐seq integratively. We found a total of 17 genes that tended to bind to IGF2BP2 among the 1124 DEGs, as shown in the Venn diagram (Figure 4E). However, we found no significant correlation between IGF2BP2‐binding genes and DEGs statistically (*P* > 0.05). Altogether, we concluded that most DEGs were not attributed to the direct binding to IGF2BP2.

### 3.5 IGF2BP2 regulates abundant AS events through functionally RNA binding

**FIGURE 5:**
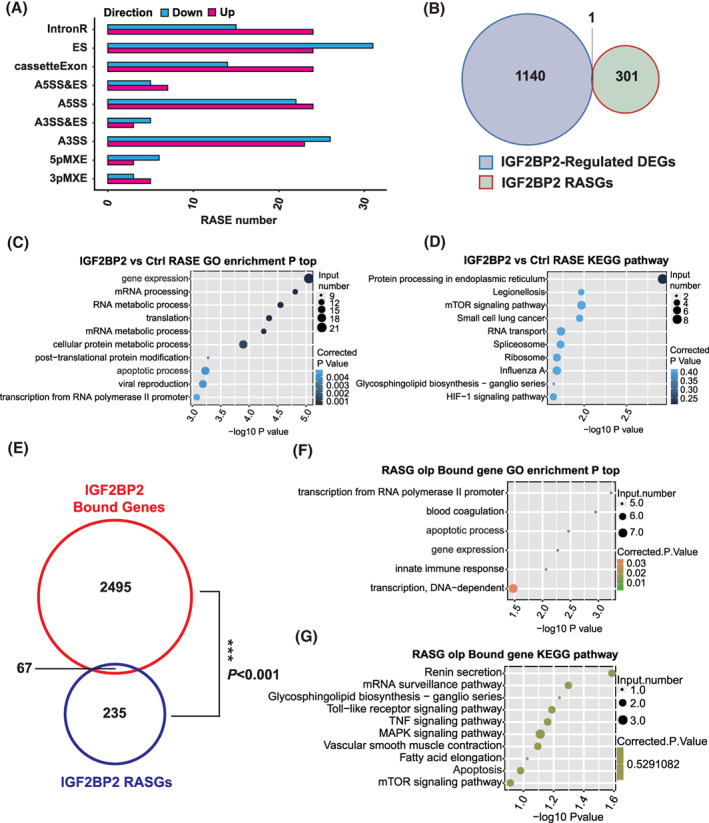
Insulin‐like growth factor 2 mRNA‐binding protein 2 (IGF2BP2)‐regulated alternative splicing events in KGN cells. (A) Classification of IGF2BP2‐regulated alternatively spliced events. (B) The overlap between IGF2BP2‐regulated differentially expressed genes and regulated alternative splicing genes (RASGs). (C) The top 10 biological processes associated with IGF2BP2‐regulated alternatively spliced genes using a GO enrichment analysis. (D) The top 10 pathways associated with IGF2BP2‐regulated alternatively spliced genes using a KEGG analysis. (E) Overlap between binding genes and RASGs. (F) Bubble plot showing the biological processes of the overlapped genes between binding genes and RASGs using a GO enrichment analysis. (G) Bubble plot showing the top KEGG pathways of the overlapped genes between binding genes and RASGs

In order to shed light on the role of IGF2BP2 in AS regulation, we used the uniquely mapped reads from the transcriptome sequencing data to explore IGF2BP2‐dependent AS events in KGN cells. The splice reads from IGF2BP2 OE and control KGN cells were mapped to the reference genome, and 367,321 annotated exons (56.53% of total annotated ones) were detected (Table S5). AS events were identified from the splice junctions, we identified 11,557 known ASEs and 29,625 novel ASEs from our analysis (Tables S6–S8). To select high‐confidence IGF2BP2 regulated alternative splicing events (RASEs), a custom pipeline was used to compare the changes in the AS ratio between IGF2BP2 OE and control cells at a cut‐off of *P*‐value < 0.05 and a changes of the AS ratio ≥0.15. Under this condition, a total of 340 RASEs were identified, including 106 intron‐retention (IR) and 234 non‐IR (NIR) RASEs. Furthermore, we also classified these high‐confidence RASEs, of which the majority included intron retention (IR, 106 events), exon skipping (ES, 55 events), cassette exon (CE, 38 events), alternative 5′splice site (A5SS, 46 events), and alternative 3′ splice site (A3SS, 49 events) (Figure 5A). These results suggested that IGF2BP2 was able to globally regulate ASEs in KGN cells. After mapping and counting, a total of 302 genes contributing to these RASEs were defined as IGF2BP2‐regulated alternative splicing genes (RASGs). There were different alternatively spliced genes between IGF2BP2 overexpression and control samples, but only one was found to be significantly regulated at a transcript level in RASGs (Figure 5B). Therefore, the observed changes in AS events cannot be simply attributed to an up‐ or downregulated transcription.

To explore the potential function of RASGs, GO terms analysis was conducted. The GO biological processes analysis suggested that the genes regulated by IGF2BP2 were highly enriched for gene expression, mRNA processing and apoptosis (Figure 5C). Similarly, the enriched KEGG pathways were related to protein processing in the endoplasmic reticulum, legionellosis, mTOR signalling pathway (Figure 5D). Hence, based on our functional analysis, it could be concluded that IGF2BP2 influenced the expression of certain genes by regulating AS in KGN cells.

To further explore these AS genes directly regulated by IGF2BP2, we performed an integrated analysis of the overlapped genes between bound genes and RASGs. A small fraction of the IGF2BP2‐regulated alternatively spliced genes (67/235) overlapped with the set of IGF2BP2‐bound genes (Figure 5E), which suggested that IGF2BP2 binding might affect some AS events in certain RNA targets. Then, we performed GO and KEGG pathway enrichment analyzes to explore the biological functions of these overlapped genes. In this case, the top hits obtained from the both analyzes were apoptotic process (Figure 5F,G). Among these 67 IGF2BP2‐regulated genes, we validated four splicing events including MBD3, FN1, TFDP1 and MKNK2 by qPCR, which were confirmed to be 5pMXE, ES, A3SS and A3SS, respectively (Figure S3A–D). These results were consistent with our RNA‐seq data. Collectively, we demonstrated that IGF2BP2 can regulate abundant AS events through functionally RNA binding in KGN cells.

### 3.6 IGF2BP2 regulates NFIC AS and further promotes GCs proliferation

**FIGURE 6:**
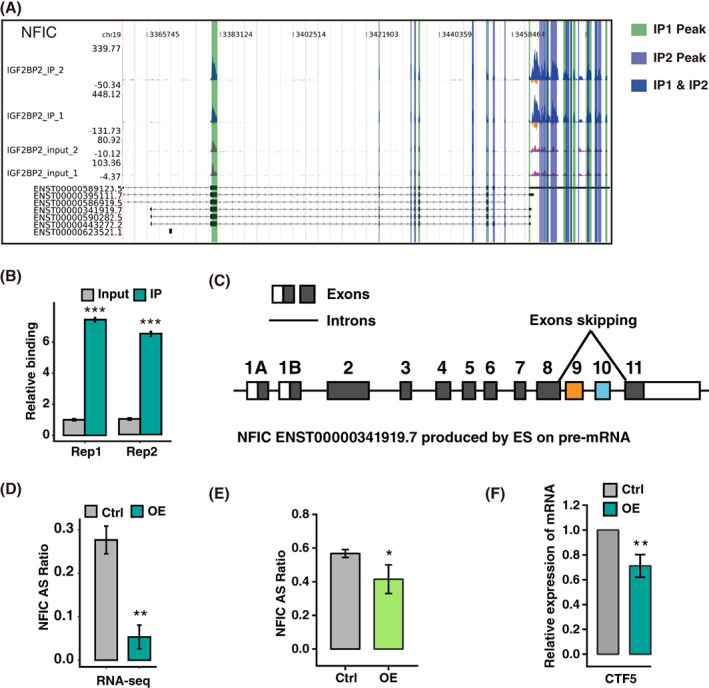
Insulin‐like growth factor 2 mRNA‐binding protein 2 (IGF2BP2) overexpression (OE) regulates alternative splicing of nuclear factor 1 C‐type (NFIC). (A) IGF2BP2 binds to the mRNAs of NFIC. Genome visualization showing IGF2BP2‐regulated ASEs and relative binding on the left and right panels, respectively. The peak ranges were highlighted with purple or green colours. (B) Quantification of NFIC binding using RNA immunoprecipitation sequencing data. ***P < 0.001 (C) Schematic diagram of the NFIC AS (ENST00000341919.7). Boxes represent exons, lines between exons represent introns. (D) The altered ratios of ASEs in RNA‐seq were plotted. **P < 0.01. (E) RT‐qPCR validation of AS of NFIC in IGF2BP2 OE KGN cells. *P < 0.05. (F) The relative expression of CTF5 was detected using RT‐qPCR following IGF2BP2 OE in KGN cells. **P < 0.01. ASEs, alternative splicing events; ES, exon skipping. Error bars represent the mean ± SEM

To confirm the potential regulators of IGF2BP2‐binding mRNAs from the RIP‐seq data and RASGs, we identified all the peaks shared by the two bindings. RNA component of signal recognition particle 7SL1 (RN7SL1), parathymosin (PTMS), NFIC, FOS like 2 (FOSL2), vasodilator stimulated phosphoprotein (VASP) and nuclear receptor subfamily 5 group A member 1 (NR5A1) were selected by ranking the max‐height peaks in a descending order. Among these potential binding genes, NFIC, FOSL2, NR5A1 and VASP were further verified by RIP‐PCR validation (Figure 6A,B; Figure S4A–C). Dysregulation of NFIC was recently reported to be involved in cell proliferation, migration, tumour invasion.^25^, ^26^ Specifically, IGF2BP2 bound to the CDS and 3′UTR region of NFIC. The splice variant of AS was formed by skipping exons 9 and 10 in the NFIC gene and generated the protein CTF5 (ENST00000341919.7) (Figure 6C), which exhibited the strongest transcriptional activation activity in the NFIC family.^27^ To further confirm the specific AS modulation, we calculated AS ratio in the RNA‐seq data and performed RT‐qPCR validation. The ratio of AS was both significantly decreased after IGF2BP2 OE in the KGN cells (Figure 6D,E). Additionally, we confirmed that it was consistent with the expression pattern in PCOS GCs (Figure S4D). To further verify the role of IGF2BP2 in regulating CTF5 expression, we detected the mRNA of CTF5 after overexpressing IGF2BP2. RT‐qPCR assay showed that enforced expression of IGF2BP2 decreased the expression of CTF5 (Figure 6F). Collectively, these results implied that IGF2BP2 OE decreased the expression of CTF5 in KGN cells.

**FIGURE 7:**
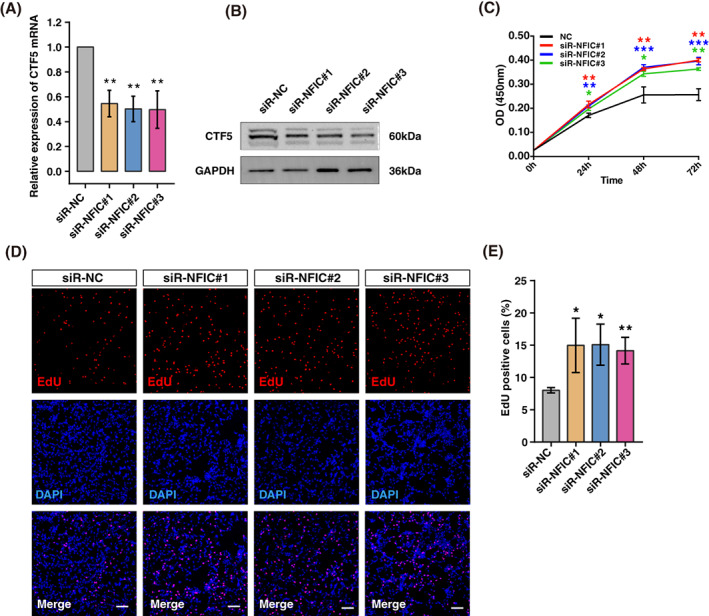
Knockdown of nuclear factor 1 C‐type (NFIC) promotes cell proliferation in KGN cells. (A) The relative expression of CTF5 mRNA is detected following NFIC knockdown in KGN cells. **P < 0.01. (B) Western blot analysis of CTF5 following NFIC knockdown. (C) KGN cells were transfected with siRNA‐NC or siRNA‐NFIC#1/ #2/ #3 for 24, 48, or 72 h, and cell viability was determined by the CCK‐8 assay. *P < 0.05, **P < 0.01, ***P < 0.001. (D) EdU staining of NFIC knockdown cells. Nuclei were stained by using DAPI. EdU positive cells, red; cell nuclei, blue; the data shown were representative of three independent experiments with similar results. Scale bar, 100 μm. (E) Statistics of EdU positive cells quantified by counting the cells with fluorescent signal using the software Image J. Experiments were performed in triplicate. *P < 0.05, **P < 0.01

Given that CTF5 functions as a regulator in cell proliferation, we next investigated whether CTF5 was responsible for the IGF2BP2 OE‐induced proliferation in KGN cells. We knocked down NFIC using siRNAs and it was significantly reduced at both transcriptional and translational levels (Figure 7A,B). As expected, CCK‐8 showed that knockdown of NFIC significantly improved viability of KGN cells (Figure 7C). Meanwhile, EdU assay displayed higher ratio of EdU positive cells in NFIC knockdown group compared to negative control, which exactly indicated that NFIC silencing promoted cell proliferation (Figure 7D,E). Accordingly, these findings implied that IGF2BP2 OE promoted proliferation through regulating AS of NFIC.

## 4 DISCUSSION

PCOS is a common endocrinopathy with complex pathophysiology that can result in anovulatory infertility in women.^28^ It is reported that excessive proliferation of GCs is one of the main causes of ovarian dysfunction, which contributes to PCOS.^29^ Recently, increasing evidence has suggested that the splicing patterns are aberrant in abnormal follicular development and to ovarian disorders.^12^, ^30^ So far, the molecular mechanism associating AS events in GCs in PCOS is still unclear. In the present study, we performed RNA‐seq and RIP‐seq to comprehensively study IGF2BP2‐mediated AS on a genome‐wide scale and attempted to explore the specific roles of IGF2BP2 in causing abnormal cell proliferation. Integrated analysis showed that IGF2BP2 was able to regulate AS events, and the overexpression of IGF2BP2 could promote cell proliferation through regulating NFIC AS.

IGF2BP2, belonging to a conserved family of RBPs, is associated with a large number of diseases, including diabetes, acute myelocytic leukaemia,^31^ and colorectal carcinoma,^32^ for its abnormal energy expenditure and life span in pathological conditions.^33^ In a previous study, IGF2BP2 was highly expressed in the GCs of women with PCOS and it was possible to demonstrate that it serves as an HMGA2 target gene and can promote the proliferation of GCs by the HMGA2/IGF2BP2 pathway.^8^ Here, we uncovered the binding profile of IGF2BP2 in KGN cells via RNA‐seq and found that IGF2BP2 preferentially binds to the 3′ and 5′UTRs of mRNAs. IGF2BPs were a distinct family of *N* ^6^‐methyladenosine (m^6^A) readers that target thousands of mRNA transcripts through recognizing the consensus GG(m^6^A)C sequence, with binding sites enriched in m^6^A motif ‘GGAC’.^24^ Besides the previously confirmed GGAC motif, we also found a novel GAAG motif. GGAC's binding sites were enriched at the 5′ UTRs while the GAAG motif was enriched at the 3′ UTRs. The recognition of both 5′ and 3′ splice sites in pre‐mRNA was an essential event in the AS decision. Nevertheless, when integrated with RNA‐seq data, the majority of alternatively splicing genes that are regulated by IGF2BP2 showed no significant differences in transcription levels. Therefore, we inferred that alterations in ASEs could not be simply attributed to transcriptional regulation.

Among a set of IGF2BP2 regulated‐RASGs which simultaneously require IGF2BP2‐RNA binding, we found NFIC, playing important roles during normal development and associated with developmental abnormalities in humans,^34^ might be a potential pathogenic gene. We clarified that IGF2BP2 served as a splicing factor to broadly regulate ASEs and inhibited the production of CTF5 by regulating exons 9 and 10 skipping in *NFIC*. Transcripts of each NFIC are differentially spliced, yielding distinct proteins from a single gene.^35^ CTF5, a splicing variant of NFIC, is the strongest transcriptional activator of the NFIC family.^27^ Previously, CTF5 was reported to participate in the MCPIP1‐mediated antiproliferative effect by transcriptionally repressing cyclin D1.^36^ In our study, when blocking the production of CTF5 by knocking down NFIC, the viability and proliferation were obviously increased in KGN cells, which suggested that IGF2BP2 may promote GC proliferation by regulating the AS of NFIC. The HMGA2/IGF2BP2 pathway is activated and induces the expression of CCND2 and SERBP1 in PCOS patients.^8^ As a multifunctional regulator, IGF2BP2 is probably involved in the pathogenesis of PCOS through different pathways and its upstream regulator, HMGA2, is attributed to initate the dysfunction of cell proliferation, which is also related to T2DM and obesity.^37^, ^38^ Therefore, the CTF5 mediated by IGF2BP2 might play a role in the development of PCOS. However, the exact roles of CTF5 still need to be elucidated more precisely.

## 5 CONCLUSIONS

In conclusion, we deciphered IGF2BP2‐interacting transcripts and global transcriptome together with AS by RIP‐seq and RNA‐seq in KGN cells. Notably, our study is the first to demonstrate IGF2BP2 promotes GC proliferation via regulating AS of NFIC in PCOS. Therefore, our findings provide new insights into the study on pathogenesis of PCOS and suggest opportunities for new diagnostic marker and therapeutic target for PCOS.

## CONFLICT OF INTEREST

The authors wish to declare that they have no conflict of interest.

## AUTHOR CONTRIBUTIONS

Feiyan Zhao and Liang Wu performed most of the experiments. Qin Wang collected clinical samples. Xuehan Zhao and Tong Chen analysed data. Long Yan and Xiaokui Yang designed the most experiments. Chenghong Yin and Xiaokui Yang supervised the project. Feiyan Zhao wrote the manuscript with input from all co‐authors.

## Supporting information


**Figure S1** Schematic of granulosa cells collection from control women and PCOS patients when undergoing oocytes retrieval.

**Figure S2** (A) An extension of Figure 2G, the relative expression of DEGs (THEMIS2, FAM133B, STAB2) and qPCR validation. Error bars represent the mean ± SEM. **P <0.01, ***P <0.001.

**Figure S3** IGF2BP2 regulates alternative splicing of MBD3. (A), FN1(B), TFDP1 (C) and MKNK2 (D), with the schematic diagrams depict ASEs structure, AS1 (purple line) and AS2 (green line), alternative 5pMXE events in MBD3, alternative ES events in FN1, alternative A3SS events in TFDP1, alternative A3SS events in MKNK2. The constitutive exon sequences are denoted by the black boxes, intron sequences by the horizontal line (right panel, top), while the alternative exons are illustrated by the red boxes and the introns by the purple boxes. RNA‐seq quantification and RT‐qPCR validation of ASEs are shown at the bottom of the right panel. Error bars represent the mean ± SEM. *P <0.05, **P <0.01, ***P <0.001.

**Figure S4** IGF2BP2 binds to the mRNAs of the genes NR5A1. (A), VASP (B) and FOSL2 (C), genome visualization showing IGF2BP2‐regulated ASEs and relative binding on the left and right panels, respectively. The peak ranges were highlighted with purple or green colours. (D) RT‐qPCR validation of NFIC AS in GCs of PCOS. *P <0.05

**Table S1** RT‐qPCR primers for gene expression qualification.

**Table S2** Summary of sample names, description, the RNA‐seq sequencing information and mapping results in each sample

**Table S3** Human genes detection level by different FPKM cut off

**Table S4** Mapping of clean reads on the reference genome

**Table S5** Exons detection results in RNA‐seq data

**Table S6** Splicing_Junction_analysis_of_samples_from_RNA‐seq_data

**Table S7** All_known_AS_events_detected_from_all_samples

**Table S8** Novel_AS_events_detected_from_all_samples

**Appendix S1**: Supplementary Information

## Data Availability

The RNA‐seq and RIP‐seq data produced in this work was deposited in NCBI's Gene Expression Omnibus and can be accessed through GEO series accession (GSE191031).

## References

[1] Tang L‐T , Ran X‐Q , Mao N , et al. Analysis of alternative splicing events by RNA sequencing in the ovaries of Xiang pig at estrous and diestrous. Theriogenology. 2018;119:60‐68. 29982137 10.1016/j.theriogenology.2018.06.022

[2] Stener‐Victorin E , Deng Q . Epigenetic inheritance of polycystic ovary syndrome ‐ challenges and opportunities for treatment. Nat Rev Endocrinol. 2021;17(9):521‐533. 34234312 10.1038/s41574-021-00517-x

[3] Azziz R , Carmina E , Chen Z , et al. Polycystic ovary syndrome. Nat Rev Dis Primers. 2016;2:16057. 27510637 10.1038/nrdp.2016.57

[4] Puttabyatappa M , Padmanabhan V . Ovarian and extra‐ovarian mediators in the development of polycystic ovary syndrome. J Mol Endocrinol. 2018;61(4):R161‐R184. 29941488 10.1530/JME-18-0079 PMC6192837

[5] Franks S , Stark J , Hardy K . Follicle dynamics and anovulation in polycystic ovary syndrome. Hum Reprod Update. 2008;14(4):367‐378. 18499708 10.1093/humupd/dmn015

[6] Vilarino‐Garcia T , Perez‐Perez A , Santamaria‐Lopez E , Prados N , Fernandez‐Sanchez M , Sanchez‐Margalet V . Sam68 mediates leptin signaling and action in human granulosa cells: possible role in leptin resistance in PCOS. Endocr Connect. 2020;9(6):479‐488. 32375121 10.1530/EC-20-0062 PMC7354740

[7] Liu X , Sun C , Zou K , et al. Novel PGK1 determines SKP2‐dependent AR stability and reprograms granular cell glucose metabolism facilitating ovulation dysfunction. EBioMedicine. 2020;61:103058. 33096483 10.1016/j.ebiom.2020.103058 PMC7581881

[8] Li M , Zhao H , Zhao SG , et al. The HMGA2‐IMP2 pathway promotes granulosa cell proliferation in polycystic ovary syndrome. J Clin Endocrinol Metab. 2019;104(4):1049‐1059. 30247605 10.1210/jc.2018-00544 PMC6753588

[9] Baralle FE , Giudice J . Alternative splicing as a regulator of development and tissue identity. Nat Rev Mol Cell Biol. 2017;18(7):437‐451. 28488700 10.1038/nrm.2017.27 PMC6839889

[10] Soucek P , Reblova K , Kramarek M , et al. High‐throughput analysis revealed mutations' diverging effects on SMN1 exon 7 splicing. RNA Biol. 2019;16(10):1364‐1376. 31213135 10.1080/15476286.2019.1630796 PMC6779402

[11] Wang E , Lu SX , Pastore A , et al. Targeting an RNA‐binding protein network in acute myeloid leukemia. Cancer Cell. 2019;35(3):369‐384.e7. 30799057 10.1016/j.ccell.2019.01.010 PMC6424627

[12] Wang F , Pan J , Liu Y , et al. Alternative splicing of the androgen receptor in polycystic ovary syndrome. Proc Natl Acad Sci U S A. 2015;112(15):4743‐4748. 25825716 10.1073/pnas.1418216112 PMC4403157

[13] Nielsen J , Christiansen J , Lykke‐Andersen J , Johnsen AH , Wewer UM , Nielsen FC . A family of insulin‐like growth factor II mRNA‐binding proteins represses translation in late development. Mol Cell Biol. 1999;19(2):1262‐1270. 9891060 10.1128/mcb.19.2.1262 PMC116055

[14] Xu Y , Wang L , He J , et al. Prevalence and control of diabetes in Chinese adults. JAMA. 2013;310(9):948‐959. 24002281 10.1001/jama.2013.168118

[15] Cao J , Mu Q , Huang H . The roles of insulin‐like growth factor 2 mRNA‐binding protein 2 in cancer and cancer stem cells. Stem Cells Int. 2018;2018:4217259. 29736175 10.1155/2018/4217259 PMC5874980

[16] Ruchat S‐M , Elks CE , Loos RJF , et al. Association between insulin secretion, insulin sensitivity and type 2 diabetes susceptibility variants identified in genome‐wide association studies. Acta Diabetol. 2009;46(3):217‐226. 19082521 10.1007/s00592-008-0080-5

[17] Hammer NA , Hansen T , Byskov AG , et al. Expression of IGF‐II mRNA‐binding proteins (IMPs) in gonads and testicular cancer. Reproduction. 2005;130(2):203‐212. 16049158 10.1530/rep.1.00664

[18] Rotterdam ESHRE/ASRM‐Sponsored PCOS Consensus Workshop Group . Revised 2003 consensus on diagnostic criteria and long‐term health risks related to polycystic ovary syndrome (PCOS). Hum Reprod. 2004;19(1):41‐47. 14688154 10.1093/humrep/deh098

[19] Shi FT , Cheung AP , Klausen C , Huang HF , Leung PC . Growth differentiation factor 9 reverses activin A suppression of steroidogenic acute regulatory protein expression and progesterone production in human granulosa‐lutein cells. J Clin Endocrinol Metab. 2010;95(10):E172‐E180. 20660033 10.1210/jc.2010-0477

[20] Geng X , Zhao J , Huang J , et al. lnc‐MAP3K13‐7:1 inhibits ovarian GC proliferation in PCOS via DNMT1 downregulation‐mediated CDKN1A promoter hypomethylation. Mol Ther. 2021;29(3):1279‐1293. 33212300 10.1016/j.ymthe.2020.11.018 PMC7934583

[21] Wu ZH , Yue JX , Zhou T , Xiao HJ . Integrated analysis of the prognostic values of RNA‐binding proteins in head and neck squamous cell carcinoma. Biofactors. 2021;47(3):478‐488. 33651487 10.1002/biof.1722

[22] Kim D , Pertea G , Trapnell C , Pimentel H , Kelley R , Salzberg SL . TopHat2: accurate alignment of transcriptomes in the presence of insertions, deletions and gene fusions. Genome Biol. 2013;14(4):R36. 23618408 10.1186/gb-2013-14-4-r36 PMC4053844

[23] Biswas J , Patel VL , Bhaskar V , Chao JA , Singer RH , Eliscovich C . The structural basis for RNA selectivity by the IMP family of RNA‐binding proteins. Nat Commun. 2019;10(1):4440. 31570709 10.1038/s41467-019-12193-7 PMC6768852

[24] Huang H , Weng H , Sun W , et al. Recognition of RNA N‐methyladenosine by IGF2BP proteins enhances mRNA stability and translation. Nat Cell Biol. 2018;20(3):285‐295. 29476152 10.1038/s41556-018-0045-z PMC5826585

[25] Xu G , Zhang Y , Li N , et al. LBX2‐AS1 up‐regulated by NFIC boosts cell proliferation, migration and invasion in gastric cancer through targeting miR‐491‐5p/ZNF703. Cancer Cell Int. 2020;20:136. 32351330 10.1186/s12935-020-01207-w PMC7183605

[26] Liang X , Gao J , Wang Q , Hou S , Wu C . ECRG4 represses cell proliferation and invasiveness via NFIC/OGN/NF‐kappaB signaling pathway in bladder cancer. Front Genet. 2020;11:846. 32922434 10.3389/fgene.2020.00846 PMC7456849

[27] Wenzelides S , Altmann H , Wendler W , Winnacker EL . CTF5—A new transcriptional activator of the NFI/CTF family. Nucleic Acids Res. 1996;24(12):2416‐2421. 8710515 10.1093/nar/24.12.2416 PMC145930

[28] Hoeger KM , Dokras A , Piltonen T . Update on PCOS: consequences, challenges, and guiding treatment. J Clin Endocrinol Metab. 2021;106(3):e1071‐e1083. 33211867 10.1210/clinem/dgaa839

[29] Yang H , Xie Y , Yang D , Ren D . Oxidative stress‐induced apoptosis in granulosa cells involves JNK, p53 and Puma. Oncotarget. 2017;8(15):25310‐25322. 28445976 10.18632/oncotarget.15813 PMC5421932

[30] Hu Y , Ouyang Z , Sui X , et al. Oocyte competence is maintained by m(6)A methyltransferase KIAA1429‐mediated RNA metabolism during mouse follicular development. Cell Death Differ. 2020;27(8):2468‐2483. 32094512 10.1038/s41418-020-0516-1 PMC7370231

[31] He X , Li W , Liang X , et al. IGF2BP2 overexpression indicates poor survival in patients with acute myelocytic leukemia. Cell Physiol Biochem. 2018;51(4):1945‐1956. 30513526 10.1159/000495719

[32] Li T , Hu PS , Zuo Z , et al. METTL3 facilitates tumor progression via an m(6)A‐IGF2BP2‐dependent mechanism in colorectal carcinoma. Mol Cancer. 2019;18(1):112. 31230592 10.1186/s12943-019-1038-7 PMC6589893

[33] Ikemoto A , Sato DX , Makino T , Kawata M . Genetic factors for short life span associated with evolution of the loss of flight ability. Ecol Evol. 2020;10(12):6020‐6029. 32607209 10.1002/ece3.6342 PMC7319159

[34] Chen KS , Lim JWC , Richards LJ , Bunt J . The convergent roles of the nuclear factor I transcription factors in development and cancer. Cancer Lett. 2017;410:124‐138. 28962832 10.1016/j.canlet.2017.09.015

[35] Gronostajski RM . Roles of the NFI/CTF gene family in transcription and development. Gene. 2000;249(1–2):31‐45. 10831836 10.1016/s0378-1119(00)00140-2

[36] Chen F , Wang Q , Yu X , et al. MCPIP1‐mediated NFIC alternative splicing inhibits proliferation of triple‐negative breast cancer via cyclin D1‐Rb‐E2F1 axis. Cell Death Dis. 2021;12(4):370. 33824311 10.1038/s41419-021-03661-4 PMC8024338

[37] Markowski DN , Thies HW , Gottlieb A , Wenk H , Wischnewsky M , Bullerdiek J . HMGA2 expression in white adipose tissue linking cellular senescence with diabetes. Genes Nutr. 2013;8(5):449‐456. 23881689 10.1007/s12263-013-0354-6 PMC3755135

[38] Anand A , Chada K . In vivo modulation of Hmgic reduces obesity. Nat Genet. 2000;24(4):377‐380. 10742101 10.1038/74207

