# Supplementary material for: Insulin‐like growth factor 2 mRNA‐binding protein 2‐regulated alternative splicing of nuclear factor 1 C‐type causes excessive granulosa cell proliferation in polycystic ovary syndrome
Source: Cell Prolif. 2022 Mar 16;55(4):e13216. doi: 10.1111/cpr.13216 (PMC9055906; doi:10.1111/cpr.13216)
Supplement: Supplementary file 13 — Appendix S1: Supplementary Information [file CPR-55-e13216-s004.docx]

Supporting information for Materials and methods

1. Western blotting

WB was performed as described by Yang et al.^1^. Cells were lysed in ice-cold RIPA lysis buffer (P0013B, Beyotime, China) containing a protease inhibitor cocktail (Roche, Basel, Switzerland) and phosphatase inhibitor (Roche). Then, 25 μg protein was subjected to (SDS-PAGE) followed by electro transfer onto PVDF membranes. After blocking with 5% skim milk for 1 h, the membranes were incubated with primary antibodies against IGF2BP2 (1:2000; 11601-1-AP, Proteintech), CTF5 (1:1000; K009930P; Solarbio, China) or GAPDH (1:2000; ab22556; Abcam) overnight at 4 °C, followed by incubation with horseradish peroxidase-conjugated secondary antibodies (Zhongshan Golden Bridge Biotechnology Co., Ltd., Beijing, China) for 1 h at room temperature (RT). Signals were developed using the enhanced chemiluminescence system (Piece, Rockford, IL, USA). Densitometric quantification was performed using Scion Image software (Scion Corp., Frederick, MD, USA).

2. Cloning and Plasmid Construction

Primer pairs used for Hot Fusion were designed with CE Design V1.04. Each of the primer comprises of a specific gene fragment sequence and a 17–30 bp sequence of the pIRES-hrGFP-1a vector. The forward (F) and reverse (R) sequences of the primers used were:

F-primer: AGCCCGGGCGGATCCGAATTCATGATGAACAAGCTTTACATCG

R-primer: GTCATCCTTGTAGTCCTCGAGCTTGCTGCGCTGTGAGGC

The pIRES-hrGFP-1a vector was digested by EcoRI and XhoI (NEB) at 37℃ for 2h-3h. After digestion, we ran the vector on a 1.0% agarose gel and purified using a Qiagen column kit. Total RNA was isolated from KGN cells with Trizol. Purified RNA was transcribed for cDNA by oligo dT primer, after which the insert fragment was synthesized by PCR amplification. We added the linearized vector (i.e. digested with EcoRI and XhoI) and the PCR insert to a PCR microtube for ligation with ClonExpress® II One Step Cloning Kit (Vazyme, Nanjing, China). The plasmids were introduced into an *Escherichia coli* strain by chemical transformation, and the cells were plated onto LB agar plates containing 1µL/ml ampicillin and incubated overnight at 37℃. The colonies were screened by colony PCR (28 cycles) with universal primers (located on the backbone vector). The insert sequence was confirmed by Sanger sequencing.

3. Cell Counting Kit-8 (CCK-8) assay

KGN cells were transiently transfected with the corresponding plasmids and siRNAs. The next day the transfected cells (1.5×10^4^/well) were plated in 96-well plates in triplicates. Cell viability was assessed at 24, 48 and 72 h, respectively. CCK-8 reagent (10 μL/well) was added to each well and incubated at 37 °C in dark for 4 h. The absorbance at 450 nm was examined with the SpectraMax Absorbance Reader (Molecular Devices, USA)

4. 5-ethynyl-2'-deoxyuridine (EdU) assay

The KGN cells were transfected with IGF2BP2-overexpressing plasmid or NFIC siRNA, incubated with 5-ethynyl-20-deoxyuridine (EdU) (C10310-1, Ribobio, Guangzhou, China) for 2 h, and processed according to the manufacturer’s instruction. After three washes with phosphate-buffered saline (PBS), the cells were fixed in 4% paraformaldehyde fix solution (PFA) for 30 min at RT and then treated with 50 μl of 2 mg/ml glycine. PBS with 0.5% TritonX-100 was used to permeabilize the cells for 10 min. The cells were incubated in 1×Apollo®567 reaction cocktail for 30 min after three washes with PBS. Then, the DNA contents of the cells in each well were stained with 100 μl of 4′,6-diamidino-2-phenylindole (DAPI, 1:1000; Sigma-Aldrich) for 30 min, and images were captured under a confocal laser scanning microscope (Carl Zeiss LSM880).

5. RNA extraction and sequencing

The KGN cells were ground into a fine powder before RNA extraction. Total RNA was treated with RQ1 DNase (Promega) to remove DNA. For each sample, 0.5μg of total RNA was used for RNA-seq library preparation using the KAPA Stranded mRNA-Seq Kit for Illumina® Platforms (KK8544). Polyadenylated mRNAs were purified, fragmented and converted into double strand cDNA. Following the end repair and A tailing, the DNA fragments were ligated to the Diluted Roche Adaptor (KK8726). After purifying and size fractioning to 300-500bps, the ligated products were amplified and purified, quantified and stored at -80℃ before sequencing. The strand marked with dUTP (the 2nd cDNA strand) was not amplified, allowing for strand-specific sequencing.

The libraries were prepared for high-throughput sequencing following the manufacturer's instructions for the Illumina NovaSeq 6000 system with 150 nt paired-end sequencing.

6. RNA-Seq Raw Data Clean and Alignment

We discarded raw reads containing more than 2-N bases and then trimmed the adaptors and low-quality bases from raw sequencing reads using the FASTX-Toolkit (Version 0.0.13). We have also discarded reads shorter than 16nt. The dataset containing the clean reads was aligned to the GRch38 genome by tophat2 ^2^ allowing 4 mismatches. Uniquely mapped reads were used for gene reads number counting and FPKM calculation (fragments per kilobase of transcript per million fragments mapped) ^3^.

7. Alternative splicing analysis

The alternative splicing events (ASEs) and regulated alternative splicing events (RASEs) between the samples were defined and quantified using the ABLas pipeline as described previously ^4^. Briefly, ABLas detected ten types of ASEs based on the splice junction reads, including exon skipping (ES), alternative 5' splice site (A5SS), alternative 3' splice site (A3SS), intron retention (IR), mutually exclusive exons (MXE), mutually exclusive 5' untranslated regions (UTRs) (5pMXE), mutually exclusive 3'UTRs (3pMXE), cassette exon, A3SS & ES and A5SS & ES.

To assess RBP regulated ASEs, we evaluated the significance of the ratio alteration of AS events by performing a Student’s t-test. Those events with a significant at P-value cut-off corresponding to a false discovery rate (FDR) cut-off of 5% were considered RBP regulated ASEs.

8. Reverse transcription qPCR validation of DEGs and AS events

We performed quantitative reverse-transcription polymerase chain reaction (RT-qPCR) for some of the DEGs to elucidate the validity of the RNA-seq data. Information on the primers utilized was presented in Table S1. Real-time PCR was performed with the Step One Real Time PCR System using the SYBR Green PCR Reagents Kit (Yeasen, Shanghai, China). The RNA expression levels of all genes were normalized against the expression level of GAPDH.

Additionally, we performed an RT-qPCR assay in order to validate ASEs. Information on the primers utilized for detecting ASEs was shown in Additional file 1. To detect alternative isoforms, we used a boundary-spanning primer for the sequence encompassing the junction of a constitutive exon and an alternative exon as well as an opposing primer in a constitutive exon. The boundary-spanning primer of the alternative exon was designed according to “model exon” to detect model splicing or “altered exon” to detect altered splicing.

9. RIP-seq library preparation and sequencing

KGN cells were irradiated once for 400 mJ/cm2, ground in liquid nitrogen and lysed in ice-cold wash buffer. Cells lysis was performed in cold wash buffer (1× PBS, 0.1% SDS, 0.5% NP-40 and 0.5% sodium deoxycholate) supplemented with a 200 U/mL RNase inhibitor (Takara, Japan) and protease inhibitor cocktail (Roche) and incubated on ice for 30 min. We cleared the cell lysate by centrifugation at 10,000 rpm for 10 min at 4°C. We then added RQ I (1 U/μl, Promega, England) to a final concentration of 1 U/μl and incubated it in a water bath for 30 min at 37°C. Immediately afterwards, we added a stop solution to the lysates in order to quench DNase. The mixture was then vibrated vigorously and centrifuged at 13,000 *×* g at 4℃ for 20 min to remove cell debris. RNA digestion was then performed by MNase (EN0181, Thermo Scientific, Germany).

For immunoprecipitation, the supernatant was incubated overnight at 4 °C with 10μg IGF2BP-antibody (11601-1-AP, Proteintech, Chicago, USA) and control IgG-antibody (2729S, CST, USA). The immunoprecipitates were further incubated with protein A /G Dynabeads for 2h at 4 °C. After applying the magnet and removing the supernatant, we sequentially washed the beads with lysis buffer, high-salt buffer (250 mM Tris 7.4, 750 mM NaCl, 10 mM EDTA, 0.1% SDS, 0.5% NP-40 and 0.5% deoxycholate), and PNK buffer (50 mM Tris, 20 mM EGTA and 0.5% NP-40) twice. The beads were then resuspended in Elution buffer (50 nM Tris 8.0, 10 mM EDTA and 1% SDS) and the suspension was incubated for 20 min in a heat block at 70°C to release the immunoprecipitated RBP with crosslinked RNA before vortex. The magnetic beads were removed from the separator and the supernatant was transferred to a clean 1.5 ml microfuge tube. Proteinase K (Roche) was added to the 1% input (without immunoprecipitated）and the immunoprecipitated RBP with crosslinked RNA to a final concentration of 1.2 mg/ml, and the mixture was incubated for 120 min at 55°C. The RNA was purified with the Trizol reagent (Life technologies, USA).

cDNA libraries were prepared with the KAPA RNA Hyper Prep Kit (KK8541, KAPA, Boston, USA) according to the manufacturer’s protocol. We prepared high-throughput sequencing libraries following the manufacturer's instructions for the Illumina NovaSeq 6000 system with 150 nt paired-end sequencing.

10. Transfection of small interfering RNA

Short interfering RNAs (siRNAs) were purchased from Ribobio (Guangzhou, China). The cells (2×10^5^) were seeded into six-well plates, cultured overnight, and transfected with siRNAs using the Lipofectamine 3000 Reagent (Invitrogen) according to the manufacturer’s protocol. After transfection, the cells were incubated for 48 h before further treatment. The specific sequences of target genes were as follows:

siRNA- NFIC#1, 5’-GCTCTGCATTTCCCTACGA-3’

siRNA- NFIC#2, 5’-TGAGCATCACCGGCAAGAA-3’

siRNA- NFIC#3, 5’-CTGGAACAGGACCCAACTT-3’

11. Data Analysis

After aligning the reads to the human reference genome GRCH38 with TopHat 2, we only kept those reads that mapped uniquely for downstream analysis. The “ABLIRC” strategy was used to identify the genomic regions of IGF2BP2 binding ^4^. Reads showing an overlap of at least 1 bp were clustered as peaks. For each gene, we used computer simulation to randomly generate reads having the same number and sequence lengths as empirical reads in the peaks. The reads obtained were further mapped to the same genes to generate random max peak height from overlapping reads. We repeated the entire process 500 times and selected the peaks with heights greater than those of random max peaks (*P* < 0.05). The IP and input samples were analyzed independently via simulations, and the IP peaks overlapping with Input peaks were removed. We finally determined the IP target genes by the peaks and used the HOMER software to call the IP binding motifs ^5^.

**References**

1. Yang X, Zhou Y, Peng S, et al. Differentially expressed plasma microRNAs in premature ovarian failure patients and the potential regulatory function of mir-23a in granulosa cell apoptosis. *Reproduction.* 2012;144(2):235-244.

2. Kim D, Pertea G, Trapnell C, Pimentel H, Kelley R, Salzberg SL. TopHat2: accurate alignment of transcriptomes in the presence of insertions, deletions and gene fusions. *Genome Biol.* 2013;14(4):R36.

3. Trapnell C, Williams BA, Pertea G, et al. Transcript assembly and quantification by RNA-Seq reveals unannotated transcripts and isoform switching during cell differentiation. *Nat Biotechnol.* 2010;28(5):511-515.

4. Xia H, Chen D, Wu Q, et al. CELF1 preferentially binds to exon-intron boundary and regulates alternative splicing in HeLa cells. *Biochim Biophys Acta Gene Regul Mech.* 2017;1860(9):911-921.

5. Heinz S, Benner C, Spann N, et al. Simple combinations of lineage-determining transcription factors prime cis-regulatory elements required for macrophage and B cell identities. *Mol Cell.* 2010;38(4):576-589.
